# In *Silico* Investigation of the Pharmacological Mechanisms of Beneficial Effects of *Ginkgo biloba* L. on Alzheimer’s Disease

**DOI:** 10.3390/nu10050589

**Published:** 2018-05-10

**Authors:** Hongxiang Li, Xiaoyuan Sun, Fan Yu, Lijia Xu, Jianhua Miu, Peigen Xiao

**Affiliations:** 1 Institute of Medicinal Plant Development, Chinese Academy of Medical Sciences and Peking Union Medical College, 151 Malianwa North Road, Beijing 100193, China; aranlee@foxmail.com (H.L.); xy_sun0422@163.com (X.S.); yfan_1024@163.com (F.Y.); pgxiao@implad.ac.cn (P.X.); 2 Key Laboratory of Bioactive Substances and Resources Utilization of Chinese Herbal Medicine, Ministry of Education, 151 Malianwa North Road, Beijing 100193, China; 3 Guangxi Institute of Medicinal Plant Development, Nanning, 189 Changgang Road, Nanning 520023, China; mjh1962@vip.163.com

**Keywords:** *Ginkgo biloba* L., Alzheimer’s disease, inverse docking, systems pharmacology, dietary supplements, alternative therapies, medicine food homology

## Abstract

Based on compelling experimental and clinical evidence, *Ginkgo biloba* L. exerts a beneficial effect in ameliorating mild to moderate dementia in patients with Alzheimer’s disease (AD) and other neurological disorders, although the pharmacological mechanisms remain unknown. In the present study, compounds, their putative target proteins identified using an inverse docking approach, and clinically tested AD-related target proteins were systematically integrated together with applicable bioinformatics methods in *silico*. The results suggested that the beneficial effects of *G. biloba* on AD may be contributed by the regulation of hormone sensitivity, improvements in endocrine homeostasis, maintenance of endothelial microvascular integrity, and proteolysis of tau proteins, particularly prior to amyloid β-protein (Aβ) plaque formation. Moreover, we identified six putative protein targets that are significantly related to AD, but have not been researched or have had only preliminary studies conducted on the anti-AD effects of *G. biloba*. These mechanisms and protein targets are very significant for future scientific research. In addition, the existing mechanisms were also verified, such as the reduction of oxidative stress, anti-apoptotic effects, and protective effects against amyloidogenesis and Aβ aggregation. The discoveries summarized here may provide a macroscopic perspective that will improve our understanding of the molecular mechanism of medicinal plants or dietary supplements, as well as new clues for the future development of therapeutic strategies for AD.

## 1. Introduction

The number of patients with Alzheimer’s disease (AD) is predicted to increase exponentially during the next few decades [1]. The current therapies for AD are based on five main strategies [2]: cholinergic treatment, antiglutamatergic treatment, vitamins and antioxidants, nonsteroidal anti-inflammatory drugs (NSAIDs), and pharmacological management of neuropsychiatric symptoms. However, single targeted therapies has often been unsuccessful, due to fact that the pathogenesis and etiology of AD have not yet been completely elucidated [3]. In contrast, *Ginkgo biloba* L. has long been thought to be “multivalent” [4] and it has a definite positive effect in ameliorating mild to moderate dementia in patients with AD and other neurological disorders associated with old age [5].

*G. biloba*-related entries are all included in the Dietary Supplements chapter of the United States Pharmacopoeia National Formulary (USP41-NF36), including Ginkgo, Powdered Ginkgo Extract, Ginkgo Capsules, and Ginkgo Tablets. The leaves and seeds of *G. biloba* have been used medicinally in China for hundreds of years [5], and were approved by China’s Ministry of Health as “medicine food homology” (MFH) materials in 2002. EGb761, a standardized and well-defined product extract of *Ginkgo biloba* leaves, is currently one of the most popular herbal drugs. Thus, there is sufficient evidence of the efficacy and safety of *G. biloba*.

However, *G. biloba* contains a variety of compounds with potential synergistic effects that make it difficult to clarify its pharmacological mechanism. With the development of structural biology and computational chemistry, the exploration of medicinal plants and natural sources has moved into a new epoch. The inverse docking approach has been developed and used to facilitate the discovery of new drugs [6]. PharmMapper is a web server used to identify potential drug targets through inverse docking by matching the query compound to an in-house pharmacophore model database, with 23,236 proteins covering 16,159 druggable pharmacophore models and 51,431 ligandable pharmacophore models, as of January 2018 [7].

In this paper, we extensively employ currently available public databases and integrated bioinformatics methodologies and the inverse docking approach to describe a novel paradigm for constructing easily interpretable networks. This might enable us to reveal the pharmacological mechanism of *G. biloba* in its beneficial effects on AD.

## 2. Materials and Methods

### 2.1. Data Collection

● Candidate Compounds of *G. biloba*

In the present study, the compounds were collected from The Traditional Chinese Medicine System Pharmacology Database and Analysis Platform [8] (TCMSP, http://ibts.hkbu.edu.hk/LSP/tcmsp.php). The TCMSP provides absorptions, distribution, metabolism, and excretion (ADME)-related pharmacokinetic properties, including bioavailability (OB), drug-likeness (DL), blood–brain barrier (BBB), etc. Values OB ≥ 30% and DL ≥ 0.18 were affirmed as ADME screening criteria for candidate compounds. Some studies confirmed that under pathological conditions such as AD, EGb761 is able to cross the BBB [4], but EGb761 has a limited ability to cross the BBB under normal physiological conditions. Thus, BBB penetration may be an important factor that alters the effects of EGb761 or *G. biloba* in vivo. Therefore, we removed the BBB cutoff value of ≥−0.3 from the ADME screening criteria.

● Alzheimer’s disease associated protein targets

Information on AD-associated protein targets was identified from GeneCards [9] (http://www.genecards.org/) and the Comparative Toxicogenomics Database [10] (CTD, http://ctdbase.org/), which is a robust, publicly available database that provides comprehensive, user-friendly information on chemical-gene/protein interactions, and chemical-disease and gene-disease relationships. We also referred to the corresponding target protein’s unique UniProtKB ID in the UniProt database (http://www.uniprot.org/), composing an AD-associated target protein database. It is noteworthy that these two databases provide an expert review ranking of protein targets based on scientific research and literature, labeled by Relevance score (GeneCard) and Inference Score (CTD). A higher score indicates a higher correlation with AD.

### 2.2. Inverse Docking Analysis

The 2-dimensional (2D) and 3D structures of candidate compounds were drawn using ChemBioOffice 2012 (PerkinElmer Inc., Cambridge, MA, USA). The mol2 files (mol2) of the 3D molecular structures of 25 candidate compounds were uploaded to the PharmMapper [7] (http://lilab.ecust.edu.cn/pharmmapper/) and the *Human Protein Targets Only database* was selected for target prediction. Results include Protein Data Bank (PDB) database codes (PDBIDs), UniProtKB ID, target names, FitScores, and z’score. FitScores was adopted as the principal scoring to rank the proteins; among them, those with FitScores ≥ 4.5 were selected as the putative target proteins. The putative target proteins and AD-related target proteins were validated one by one, according to their unique UniProtKB ID, producing conditionally filtered results of AD-associated target proteins of candidate compounds.

### 2.3. Gene Ontology and KEGG Pathway Enrichment

The Database for Annotation, Visualization, and Integrated Discovery [11] (DAVID, v6.8, https://david.ncifcrf.gov/) was employed to conduct Gene Ontology (GO) and Kyoto Encyclopedia of Genes and Genomes (KEGG) pathway enrichment analysis. The *p*-value was used to examine the significance of the GO/pathway term enrichment with a modified Fisher’s exact test. The Benjamini value was used to globally correct the enrichment *p*-values of individual term members [11]. Those GO/pathway terms with a *p*-value of ≤0.05 and Benjamini value of ≤0.5 were regarded as significant and interesting.

### 2.4. Composite Network Integration

On the strength of above-mentioned target identification results, combined with Protein–Protein Interaction (PPI) data from STRING (https://string-db.org/) and pathway enrichment data from DAVID, Cytoscape 3.6.0 (Institute for Systems Biology, Seattle, WA, USA) [12] (http://www.cytoscape.org/) software was employed to construct Compound-Target (CT), Compound-Target-Disease and Compound-Group-Target-Pathway (CGTP) network models. Subsequently, engaging the NetworkAnalyzer plugin in Cytoscape, the parameters of the network topology were analyzed to get the Average Shortest Path Length (ASPL) and the Betweenness Centrality (BC), etc., and the more substantially contributing nodes were obtained. Besides this, R 3.4.3 software (R Core Team, Auckland, Tamaki-Makau-Rau, New Zealand) (https://www.r-project.org/) was employed to visualize the quantitative information.

## 3. Results

### 3.1. The Candidate Compounds and Putative Target Proteins

**Figure 1:**
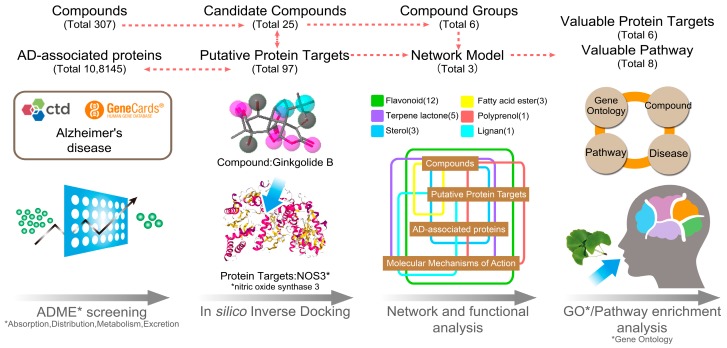
Methodology roadmap and summarized results for study.

**Figure 2:**
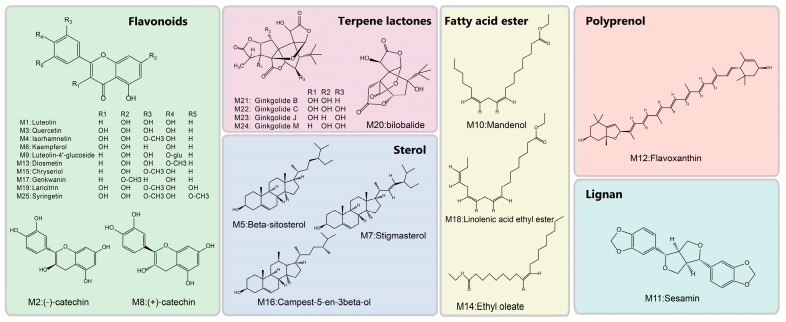
The 2D molecular structures, names, and codes of the 25 candidate compounds.

Figure 1 shows a schematic of the methodology and a summary of the results from each step. From the 307 native *G. biloba* compounds collected from the TCMSP database, 25 compounds were screened by ADME and prepared for further study as the candidate compounds as shown in Figure 2 and Supplementary Materials Table S1. The 25 compounds were divided into 6 categories: 12 flavonoids (quercetin, catechin, genkwanin, etc.), 5 terpene lactones (bilobalide, ginkgolide B, etc.), 3 sterols (beta-sitosterol, stigmasterol, and campest-5-en-3beta-ol), 3 fatty acid esters (mandenol, ethyl oleate, and linolenic acid ethyl ester), 1 polyprenol (flavoxanthin), and 1 lignan (sesamin). These compounds are the main components or active functional ingredients of *G. biloba* [4,13,14].

A total of 2500 target proteins were docked with the 25 candidate compounds. Among these target proteins, 97 were screened by ADME and named the putative target proteins. All results from the inverse docking calculation are presented in Supplementary Materials Table S2.

**Figure 3:**
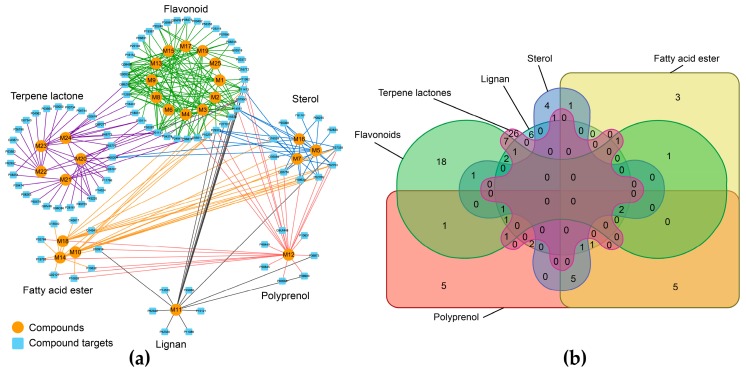
Illustration of the Compound–Target (CT) network. (**a**) Orange circles represent candidate compounds that are grouped together by structural category. The blue square represents the putative target proteins that were directly associated with candidate compounds. (**b**) The distribution of 97 putative target proteins among different compound categories, as shown in the Venn diagram.

We compared the 97 putative target proteins for commonality and properties, and the results are shown in Figure 3. Panel (a) more intuitively shows that compounds from different categories mapped to different target proteins. Moreover, panel (b) depicts a Venn diagram that clearly shows the terpene lactone group associated with 26 exclusive protein targets—approximately one-third of the protein targets. Thus, we speculated that this finding may be consistent with the observation that terpene lactones are the predominant and unique primary bioactive substances in *G. biloba*. In other words, we inferred that the specificity of the inverse docking calculation was distinct.

The gene entries related to AD were collected from the CTD and GeneCards databases. As a result, 21,249 and 7262 gene entries were collected from each database, respectively. The prioritized Inference Scores for the corresponding annotations are listed in descending order in Supplementary Materials Tables S3-1 and S4-1. The corresponding gene entries were converted into 108,145 and 58,432 UniProtKB IDs, respectively. The results are listed in Supplementary Materials Tables S3-2 and S4-2. This procedure was initiated by matching the unique UniProtKB IDs to determine the magnitude of the correlation between the putative target proteins and AD. We adopted the arithmetic average of the two scores from the CTD and GeneCards databases as integration scores, screening the top third of the putative target proteins for MOA (Molecular Mechanisms of Action) analysis. The one-third ratio was determined after several preliminary experiments. The results are listed in Supplementary Materials Table S5.

### 3.2. Exploration of the Molecular Mechanisms of Action

**Figure 4:**
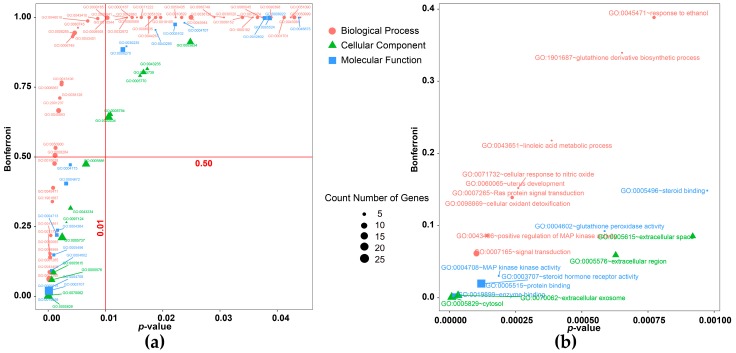
GO enrichment scatter plot for the top 30 putative target proteins. Only GO terms with a *p*-value ≤ 0.05 are shown in panel (**a**), and terms with a *p*-value ≤ 0.001 are shown in panel (**b**).

**Figure 5:**
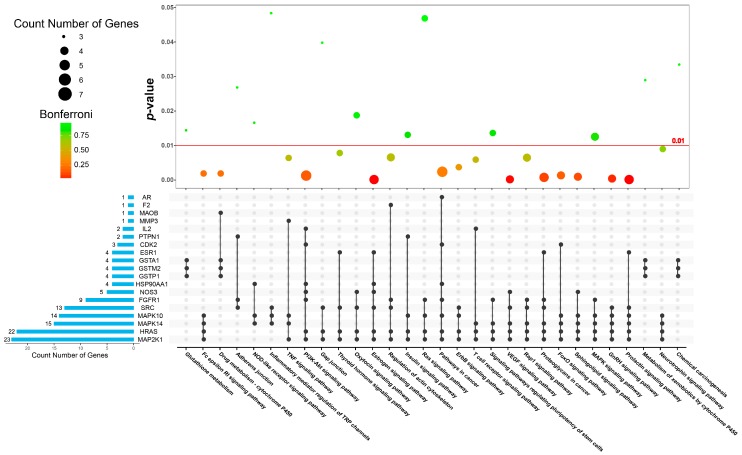
KEGG pathway enrichment bubble diagram for the top 30 putative target proteins (*p*-value ≤ 0.05).

The top 30 putative target proteins were selected based on their integration score, and GO and KEGG pathway enrichment analyses were initiated. After filtering by a parameter *p*-value cutoff of ≤0.05, 84 GO terms and 30 KEGG pathway terms were returned, as shown in Figure 4 and Figure 5 and Supplementary Materials Tables S6 and S7. A total of 84 GO terms are included: 51 for Biological Processes, 19 for Molecular Function, and 14 for Cellular Component. According to physiological function, these biological processes can be divided into 6 modules, as shown in Table 1.

In order to show the result of the KEGG pathway enrichment in an intuitive and explicit way, a bubble diagram was employed. As shown in Figure 5, *p*-values are given the highest priority, in ascending order. Dual specificity mitogen-activated protein kinase kinase 1 (MAP2K1), GTPase HRas (HRAS), mitogen-activated protein kinase 14 (MAPK14), mitogen-activated protein kinase 10 (MAPK10), and proto-oncogene tyrosine-protein kinase src (SRC) were the most frequently occurring protein targets. According to the pathogenesis of AD, these KEGG pathway terms can be divided into 5 modules, as shown in Table 2.

### 3.3. An Integrated Network Model Analysis

**Figure 6:**
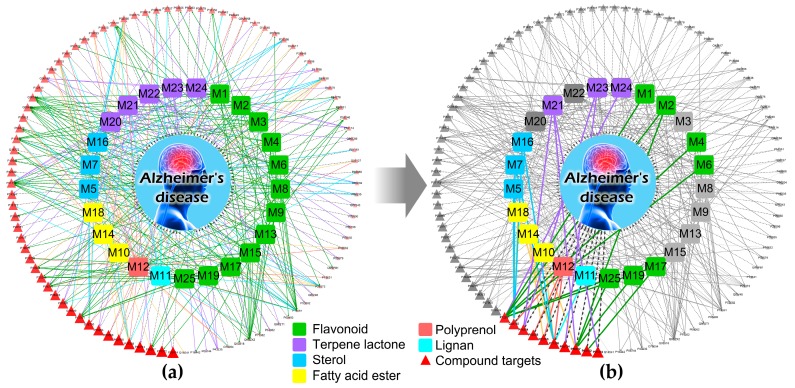
Illustration of the Compound-Target-Disease network. Rounded squares in different colors represent different groups of compounds; the code is shown on each rounded square. Red triangles represent putative target proteins, and triangles shown in darker colors and larger sizes represent greater associations with AD. All candidate compounds and putative target proteins are shown in panel (**a**). The top 10 putative target proteins and the candidate compounds directly related to them are highlighted based on the relevance score; other target proteins are grayed out in panel (**b**).

The Compound-Target-Disease network model contained 123 nodes and 369 edges, as shown in Figure 6. The top 10 putative target proteins identified based on integration scores are highlighted, and the other putative target proteins are in gray, as shown in panel (b) of Figure 6. We identified 7 flavonoids, 3 terpene lactones, 3 sterols, 3 fatty acid esters, 1 polyprenol, and 1 lignan associated with these proteins. The results suggest that flavonoids and terpene lactones may primarily contribute to the anti-AD effects of *G. biloba*. The top 10 putative target proteins associated with AD, in turn, were nitric oxide synthase (NOS3), neprilysin (NEP), beta-secretase 1 (BACE1), estrogen receptor (ESR1), amine oxidase B (MAOB), prothrombin (F2), serum albumin (ALB), transthyretin (TTR), matrix metalloproteinase-3 (MMP3), and interleukin-2 (IL2).

**Figure 7:**
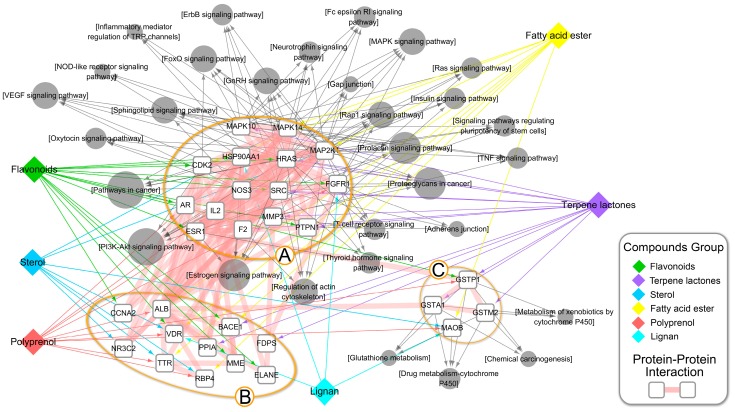
Illustration of the Compound-Group-Target-Pathway (CGTP) network. Diamonds in different colors represent different compound groups. Gray circles represent enriched KEGG pathway terms, and the size of each circle represents the number of genes that have been enriched. White rounded rectangles represent the top 30 putative target proteins. The wide pink bands represent the protein-protein interactions.

As shown in Figure 7, three subnetworks were integrated into the Compound-Group-Target-Pathway (CGTP) network, including the Protein-Protein Interaction (PPI) network, Compound-Group-Target (CGT) network, and Target-Pathway (TP) network. The PPI network is the premise and basis to obtain nodes with more substantial contributions. Nodes with a shorter ASPL and higher BC were considered as vital ones. In the PPI network, serum albumin (ALB), estrogen receptor (ESR1), and a proto-oncogene tyrosine-protein kinase (SRC) were the top three, consistent with their molecular functions of transport, connection, and signal communication. Clustering and topology approaches were utilized to identify individual variations and similarities among various protein targets. Of the top 30 putative protein targets, 3 well-organized clusters with 30 KEGG pathway terms were identified. The protein targets in cluster A were associated with 26 KEGG pathway terms, and targets in cluster C were associated with 4 KEGG pathway terms.

## 4. Discussion and Conclusions

Currently, an increasing number of people are consuming dietary supplements for health. Due to the amazing vitality induced by *G. biloba*, it has achieved therapeutic applications as a dietary supplement. Despite the fact that it has a definite positive effect in ameliorating mild to moderate dementia in patients with AD, its mechanism remains elusive. This prompted us to determine the pharmacological mechanism of *G. biloba* by inverse docking and system pharmacological approaches.

We collected 307 native *G. biloba* compounds from TCMSP, and 25 compounds were screened by ADME. Further calculations using PharmMapper identified 2500 target proteins, 97 of which were obtained by screening criteria. From the CTD and GeneCards databases, we collected 21,249 and 7262 AD-associated gene entries, respectively. The subsequent step was performed using UniProt and resulted in 108,145 and 58,432 UniprotKB IDs, respectively. Based on the integration score, the top 30 putative target proteins were selected to further explore the MOA. The functions of these putative target proteins include the following: antioxidant activity [15], protective effects on the mitochondria [16], anti-apoptotic [17], anti-inflammatory [18], protective effects on amyloidogenesis and amyloid β-protein (Aβ) aggregation [19,20], ion homeostasis [21], modulation of the phosphorylation of the tau protein [15], and induction of hormone synthesis [15]. These findings are consistent with existing experimental evidence [13,14,22]. Subsequently, 84 GO terms and 30 KEGG pathway terms were returned and classified. Finally, 3 networks were constructed and integrated.

**Figure 8:**
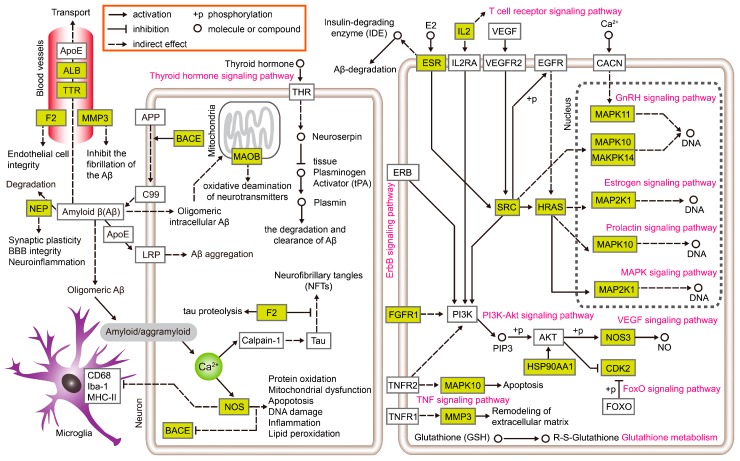
Schematic representation of the interaction effects of enriched KEGG pathway terms and putative target proteins identified in the present study. Green rectangles represent putative target proteins identified in this study. Carmine characters represent enriched KEGG pathway terms. The left panel shows potential MOAs that are directly related to Aβ synthesis, transport, degradation, and clearance. The right panel shows MOAs that are indirectly linked or irrelevant to Aβ.

In order to better understand the relationship between enriched KEGG pathways and putative target proteins, manual annotation was conducted based on KEGG pathway maps, as shown in Figure 8. The left panel shows the potential MOAs that are directly related to Aβ synthesis, transport, degradation, and clearance. The right panel shows MOAs that are indirectly linked or irrelevant to Aβ. Interestingly, the putative target proteins in the left panel are rarely enriched in the KEGG pathway terms, but the putative target proteins in the right panel are enriched in many signaling pathways. It seems that the putative target proteins in the left panel are more like “lone rangers”, while the putative target proteins in the right panel play a physiological role by interacting with other proteins; this might also be related to most of them being protein kinases. The results in Figure 7 also confirm this viewpoint, and the putative target proteins in cluster B are mostly distributed in the left panel of Figure 8. This clustering may be a possible explanation for the findings from the DAVID enrichment algorithm, but it does not hinder the process of discovering their unique roles in AD pathology. These proteins, such as Endothelial Nitric Oxide Synthase (NOS3) [23,24,25,26,27], neprilysin (NEP) [28,29], Beta-secretase (BACE) [30,31,32,33,34], Monoamine oxidases (MAOs) [35,36,37], Prothrombin (F2) [38], Serum albumin (ALB) [39], Thyroid hormone (TTR) [32], and Matrix metalloproteinase 3 (MMP3) [40], are directly or indirectly involved in Aβ synthesis, processing, aggregation, degradation and transport; the formation of neurofibrillary tangles (NFTs); and tau proteolysis. In the right panel, the hormone-related signaling pathways were on top of the KEGG pathway enrichment results. Therefore, we hypothesize that hormone-related signaling pathways may play an important role in the anti-AD effects of *G. biloba*. These signaling pathways include the (hsa04917) prolactin signaling pathway, (hsa04915) estrogen signaling pathway, (hsa04912) GnRH signaling pathway, (hsa04910) insulin signaling pathway, (hsa04921) oxytocin signaling pathway, (hsa04919) thyroid hormone signaling pathway, (hsa04722) neurotrophin signaling pathway, and (hsa04071) sphingolipid signaling pathway. Based on accumulating evidence [41,42], non-Aβ-related pathways are also an important factor in AD etiology, particularly prior to Aβ plaque formation. Prolactin [43], estrogen [44,45], oxytocin [46,47], thyroid hormone [48], and insulin [49] potentially play substantial roles in non-Aβ-related mechanisms. As for the studies on *G. biloba* related to the above-mentioned hormones, estrogen- and insulin-related studies have been extensive, but there has been less research on prolactin, oxytocin, and thyroid hormone. Based on the results of present study, it is worth further research.

As illustrated in Table 3, we identified 6 putative protein targets that were significantly related to AD, but have not been researched or have had only preliminary studies conducted on the anti-AD effects of *G. biloba*. Neprilysin (NEP), estrogen receptor (ESR), Prothrombin (F2), Serum albumin (ALB), Thyroid hormone (TTR), and Matrix metalloproteinase 3 (MMP3) are significantly associated with the development of AD. These above-mentioned protein targets are highly matched with several representative compounds in *G. biloba*, but the specific actions and properties must be further verified and probed in future experiments.

In conclusion, the establishment of networks between AD-related protein targets and compounds in *G. biloba* may have important implications for elucidating the mechanisms underlying the beneficial effects of *G. biloba* on AD. Conceivably, a novel therapeutic strategy for AD may be developed from the protein targets and pathways identified in the present study. Hopefully, a novel paradigm presented in this study would help facilitate natural medicine development and the construction of a herbal compound library.

## Figures and Tables

**Table 1:** The classification results of Biological Process terms.

Category	Biological Process Terms	*p*-Value	Bonferroni	Benjamini	FDR
signal transduction	GO:0007165~signal transduction	0.0001	0.0611	0.0611	0.1472
	GO:0043406~positive regulation of MAP kinase activity	0.0001	0.0860	0.0439	0.2099
	GO:0007265~Ras protein signal transduction	0.0002	0.1388	0.0486	0.3487
	GO:0010628~positive regulation of gene expression	0.0010	0.4753	0.0774	1.4961
	GO:0045944~positive regulation of transcription from RNA polymerase II promoter	0.0248	1.0000	0.3845	31.2264
	GO:0006367~transcription initiation from RNA polymerase II promoter	0.0022	0.7597	0.1039	3.2779
	GO:0018105~peptidyl-serine phosphorylation	0.0196	1.0000	0.3428	25.4995
	GO:0018108~peptidyl-tyrosine phosphorylation	0.0023	0.7660	0.0986	3.3386
synthesis and metabolism	GO:0043651~linoleic acid metabolic process	0.0004	0.2177	0.0479	0.5724
	GO:1901687~glutathione derivative biosynthetic process	0.0007	0.3396	0.0668	0.9652
	GO:0006749~glutathione metabolic process	0.0042	0.9309	0.1632	6.0551
hormone related	GO:0043401~steroid hormone mediated signaling pathway	0.0043	0.9371	0.1588	6.2622
	GO:0030520~intracellular estrogen receptor signaling pathway	0.0357	1.0000	0.4649	41.7726
protein metabolic	GO:0006508~proteolysis	0.0102	0.9986	0.2573	14.1863
	GO:0050435~beta-amyloid metabolic process	0.0222	1.0000	0.3699	28.4455
	GO:0044267~cellular protein metabolic process	0.0176	1.0000	0.3417	23.1933
inflammatory cascade reaction	GO:0050900~leukocyte migration	0.0012	0.5319	0.0731	1.7588
	GO:0048010~vascular endothelial growth factor receptor signaling pathway	0.0068	0.9873	0.2053	9.7017
	GO:0010544~negative regulation of platelet activation	0.0137	0.9999	0.3182	18.6122
	GO:0071222~cellular response to lipopolysaccharide	0.0162	1.0000	0.3296	21.5741
	GO:0051024~positive regulation of immunoglobulin secretion	0.0188	1.0000	0.3415	24.6631
cell apoptosis	GO:0098869~cellular oxidant detoxification	0.0002	0.1388	0.0486	0.3487
	GO:0071732~cellular response to nitric oxide	0.0003	0.1520	0.0404	0.3845
	GO:0038128~ERBB2 signaling pathway	0.0019	0.7112	0.0983	2.8617
	GO:2001237~negative regulation of extrinsic apoptotic signaling pathway	0.0019	0.7112	0.0983	2.8617
	GO:0050999~regulation of nitric-oxide synthase activity	0.0440	1.0000	0.4785	48.8128
	GO:0043410~positive regulation of MAPK cascade	0.0086	0.9959	0.2399	12.0357

**Table 2:** The classification results of KEGG pathway enrichment analysis.

Category	KEGG Pathway Terms	*p*-Value	Bonferroni	Benjamini	FDR
inflammation-generating process	hsa04370: VEGF signaling pathway	0.0001	0.0083	0.0017	0.0712
	hsa04664: Fc epsilon RI signaling pathway	0.0018	0.2196	0.0205	2.0929
	hsa04660: T cell receptor signaling pathway	0.0058	0.5537	0.0524	6.6494
	hsa04668: TNF signaling pathway	0.0063	0.5827	0.0532	7.1833
	hsa04015: Rap1 signaling pathway	0.0063	0.5848	0.0504	7.2230
	hsa04621: NOD-like receptor signaling pathway	0.0166	0.9007	0.0850	17.8802
	hsa04750: Inflammatory mediator regulation of TRP channels	0.0483	0.9989	0.1571	44.1896
apoptosis	hsa04151: PI3K-Akt signaling pathway	0.0012	0.1492	0.0160	1.3682
	hsa04068: FoxO signaling pathway	0.0012	0.1581	0.0155	1.4573
	hsa04010: MAPK signaling pathway	0.0124	0.8221	0.0755	13.6915
	hsa00480: Glutathione metabolism	0.0144	0.8645	0.0768	15.6705
	hsa05205: Proteoglycans in cancer	0.0006	0.0846	0.0110	0.7506
	hsa05200: Pathways in cancer	0.0023	0.2710	0.0240	2.6595
	hsa04014: Ras signaling pathway	0.0468	0.9987	0.1559	43.0804
	hsa04012: ErbB signaling pathway	0.0036	0.3945	0.0352	4.1891
	hsa05204: Chemical carcinogenesis	0.0334	0.9908	0.1255	32.9823
hormone synthesis and transport	hsa04917: Prolactin signaling pathway	0.0000	0.0006	0.0006	0.0053
	hsa04915: Estrogen signaling pathway	0.0000	0.0032	0.0008	0.0272
	hsa04912: GnRH signaling pathway	0.0003	0.0390	0.0057	0.3386
	hsa04910: Insulin signaling pathway	0.0130	0.8356	0.0755	14.2708
	hsa04921: Oxytocin signaling pathway	0.0187	0.9256	0.0918	19.8791
	hsa04919: Thyroid hormone signaling pathway	0.0077	0.6569	0.0547	8.7191
	hsa04722: Neurotrophin signaling pathway	0.0089	0.7084	0.0598	9.9765
	hsa04071: Sphingolipid signaling pathway	0.0008	0.1077	0.0126	0.9668
	hsa04921: Oxytocin signaling pathway	0.0187	0.9256	0.0918	19.8791
drug metabolism	hsa00982: Drug metabolism-cytochrome P450	0.0018	0.2196	0.0205	2.0929
	hsa00980: Metabolism of xenobiotics by cytochrome P450	0.0290	0.9827	0.1156	29.2357
ontogeny process	hsa04540: Gap junction	0.0398	0.9963	0.1371	37.9955
	hsa04810: Regulation of actin cytoskeleton	0.0065	0.5910	0.0484	7.3404
	hsa04520: Adherens junction	0.0268	0.9765	0.1106	27.3786

**Table 3:** The mechanisms of 6 putative protein targets related to AD and the current status of research on *G. biloba*.

Protein Targets	AD-Related Mechanisms/Etiology	Docking Compounds	Research Related to *G. biloba* for Anti-AD
Neprilysin (NEP)	(1) Aβ degradation enzymes [28,29]	(+)-Catechin Diosmetin Genkwanin	No research
	(2) maintain blood–brain barrier (BBB) integrity		
	(3) participate in neuroinflammation [50]		
Estrogen receptor (ESR)	(1) upregulated insulin-degrading enzyme (IDE) [51]	Genkwanin	No research
	(2) maintaining steroid homeostasis [52]		
	(3) altering synaptic plasticity [53,54]		
	(4) participate in neurons oxidative stress-mediated injury [55]		
Prothrombin (F2)	(1) coagulation cascade and endothelial cell integrity [38]	Ginkgolide J	No research
	(2) ideal molecular-biological indicator for AD [56]		
	(3) proteolyzes the microtubule-associated protein tau [56]		
	(4) inhibits phosphorylation of tau [56]		
Serum albumin (ALB)	(1) bounded and transported Aβ, maintaining a constant concentration level in the brain [39]	Ethyl oleate Flavoxanthin	No research
	(2) Aβ excretion from the brain to the blood [57,58]		
Thyroid hormone (TTR)	(1) up-regulation of expression of neuroserpin in neurons [32]	Beta-sitosterol Stigmasterol Mandenol Ethyl oleate Flavoxanthin	No research
	(2) hyperthyroidism increases the risk of AD [59]		
Matrix metalloproteinase 3 (MMP3)	(1) main Plasma gelsolin (GSN)-degrading enzyme [40]	Ginkgolide B Ginkgolide J	No research
	(2) inhibits the fibrillation of the Aβ [60]		
	(3) a diagnostic biomarker for AD [61]		

## Supplementary Materials

The following are available online at http://www.mdpi.com/2072-6643/10/5/589/s1. Table S1: Result of 25 candidate compounds of *Ginkgo biloba*. Table S2: Inverse Docking Results of the 25 candidate compounds of *Ginkgo biloba* L. Table S3-1: AD-associated gene information collected from Comparative Toxicogenomics Database (CTD). Table S3-2: AD-associated target proteins information collected from Comparative Toxicogenomics Database (CTD). Table S4-1: AD-associated gene information collected from GeneCards. Table S4-2: AD-associated target proteins information collected from GeneCards. Table S5: Top 30 putative target proteins according to Integrated Score. Table S6: Results of Gene Ontology enrichment for top 30 putative target proteins. Table S7: Results of pathway enrichment for top 30 putative target proteins.

## Abbreviation

AD	Alzheimer’s Disease
2D	2-dimensional
ADME	absorptions, distribution, metabolism, and excretion
ALB	serum albumin
AR	Androgen receptor
ASPL	Average Shortest Path Length
Aβ	amyloid β-protein
BACE1	beta-secretase 1
BBB	blood brain barrier
BC	Betweenness Centrality
CCNA2	Cyclin-A2
CDK2	Cyclin-dependent kinase 2
CGTP	Compound-Group-Target-Pathway
CT	Compound-Target
CTD	Comparative Toxicogenomics Database
DAVID	The Database for Annotation, Visualization, and Integrated Discovery
DL	drug-likeness
ELANE	Neutrophil elastase
ERBB2	Receptor tyrosine-protein kinase erbB-2
ESR1	Estrogen receptor 1
F2	prothrombin
FDPS	Farnesyl pyrophosphate synthase
FDR	false discovery rate
FGFR1	Fibroblast growth factor receptor 1
FOXO	forkhead box O
GO	Gene Ontology
GSTA1	Glutathione S-transferase A1
GSTM2	Glutathione S-transferase Mu 2
GSTP1	Glutathione S-transferase P
HRAS	GTPase HRas
HSP90AA1	Heat shock protein HSP 90-alpha
IL2	interleukin-2
KEGG	Kyoto Encyclopedia of Genes and Genomes
MAOB	amine oxidase B
MAP2K1	Dual specificity mitogen-activated protein kinase kinase 1
MAPK	mitogen-activated protein kinase
MAPK10	mitogen-activated protein kinase 10
MAPK14	mitogen-activated protein kinase 14
MFH	medicine food homology
MMP3	matrix metalloproteinase-3
MOA	Molecular Mechanisms of Action
NEP	neprilysin
NOD	nucleotide bindingoligomerization domain
NOS3	synthase
NR3C2	Mineralocorticoid receptor
NSAIDs	nonsteroidal anti-inflammatory drugs
OB	bioavailability
PI3K	Phosphoinositide 3-kinase
PPI	Protein–Protein Interaction
PPIA	Peptidyl-prolyl cis-trans isomerase A
PTPN1	Tyrosine-protein phosphatase non-receptor type 1
RBP4	Retinol-binding protein 4
RNA	ribonucleic acid
RnRH	Gonadotropin-releasing hormone
SRC	proto-oncogene tyrosine-protein kinase src
TCMSP	The Traditional Chinese Medicine System Pharmacology Database and Analysis Platform
TNF	Tumor Necrosis Factor
TRP	Transient receptor potential
TTR	transthyretin
USP41-NF36	United States Pharmacopoeia National Formulary
VDR	Vitamin D3 receptor
VEGF	vascular endothelial growth factor

## References

[1] Selkoe D.J. (2012). Preventing Alzheimer’s disease. Science.

[2] Lleó A., Greenberg S.M., Growdon J.H. (2006). Current Pharmacotherapy for Alzheimer’s Disease. Ann. Rev. Med..

[3] Selkoe D.J. (2011). Resolving controversies on the path to Alzheimer’s therapeutics. Nat. Med..

[4] Wolffram S., Ader P., Rimbach G., Packer L., Maguire J.J., Schultz P.G., Gohil K. (2001). The in vivo Neuromodulatory Effects of the Herbal Medicine Ginkgo Biloba. Proc. Natl. Acad. Sci. USA.

[5] Howes M.J., Houghton P.J. (2012). Ethnobotanical treatment strategies against Alzheimer’s disease. Curr. Alzheimer Res..

[6] Chen Y.Z., Zhi D.G. (2001). Ligand-protein inverse docking and its potential use in the computer search of protein targets of a small molecule. Proteins Struct. Funct. Bioinform..

[7] Wang X., Shen Y., Wang S., Li S., Zhang W., Liu X., Lai L., Pei J., Li H. (2017). PharmMapper 2017 update: A web server for potential drug target identification with a comprehensive target pharmacophore database. Nucleic Acids Res..

[8] Ru J., Li P., Wang J., Zhou W., Li B., Huang C., Li P., Guo Z., Tao W., Yang Y. (2014). TCMSP: A database of systems pharmacology for drug discovery from herbal medicines. J. Cheminform..

[9] Safran M., Dalah I., Alexander J., Rosen N., Stein T.I., Shmoish M., Nativ N., Bahir I., Doniger T., Krug H. (2010). GeneCards Version 3: The human gene integrator. Database.

[10] Davis A.P., Grondin C.J., Lennon-Hopkins K., Saraceni-Richards C., Sciaky D., King B.L., Wiegers T.C., Mattingly C.J. (2015). The Comparative Toxicogenomics Database’s 10th year anniversary: Update 2015. Nucleic Acids Res..

[11] Huang D.W., Sherman B.T., Lempicki R.A. (2009). Systematic and integrative analysis of large gene lists using DAVID bioinformatics resources. Nat. Protoc..

[12] Shannon P., Markiel A., Ozier O., Baliga N.S., Wang J.T., Ramage D., Amin N., Schwikowski B., Ideker T. (2003). Cytoscape: A software environment for integrated models of biomolecular interaction networks. Genome Res..

[13] Shi C., Liu J., Wu F., Yew D.T. (2010). Ginkgo biloba Extract in Alzheimer’s Disease: From Action Mechanisms to Medical Practice. Int. J. Mol. Sci..

[14] Tapan Kumar M., Yasinalli T., Zubaidha P.K. (2014). Phytochemical and medicinal importance of Ginkgo biloba L.. Nat. Prod. Res..

[15] Ahlemeyer B., Krieglstein J. (2003). Neuroprotective effects of Ginkgo biloba extract. Cell. Mol. Life Sci..

[16] Shi C. (2009). Protective effects of Ginkgo biloba extract (EGb761) and its constituents quercetin and ginkgolide B against β-amyloid peptide-induced toxicity in SH-SY5Y cells. Chem. Biol. Interact..

[17] Smith J.V., Luo Y. (2004). Studies on molecular mechanisms of Ginkgo biloba extract. Appl. Microbiol. Biotechnol..

[18] Maclennan K.M., Darlington C.L., Smith P.F. (2002). The CNS effects of Ginkgo biloba extracts and ginkgolide B. Prog. Neurobiol..

[19] Smith J.V., Luo Y. (2003). Elevation of oxidative free radicals in Alzheimer’s disease models can be attenuated by Ginkgo biloba extract EGb 761. J. Alzheimers Dis..

[20] Bastianetto S., Ramassamy C., Doré S., Christen Y., Poirier J., Quirion R. (2000). The Ginkgo biloba extract (EGb 761) protects hippocampal neurons against cell death induced by beta-amyloid. Eur. J. Neurosci..

[21] Berrocal M., Marcos D., Sepúlveda M.R., Pérez M., Avila J., Mata A.M. (2009). Altered Ca^2+^ dependence of synaptosomal plasma membrane Ca^2+^-ATPase in human brain affected by Alzheimer’s disease. FASEB J..

[22] Yin Y., Ren Y., Wu W., Wang Y., Cao M., Zhu Z., Wang M., Li W. (2013). Protective effects of bilobalide on Aβ(25-35) induced learning and memory impairments in male rats. Pharmacol. Biochem. Behav..

[23] Doreulee N., Sergeeva O.A., Yanovsky Y., Chepkova A.N., Selbach O., Gödecke A., Schrader J., Haas H.L. (2003). Cortico-striatal synaptic plasticity in endothelial nitric oxide synthase deficient mice. Brain Res..

[24] Austin S.A., Santhanam A.V., Hinton D.J., Choi D.S., Katusic Z.S. (2013). Endothelial nitric oxide deficiency promotes Alzheimer’s disease pathology. J. Neurochem..

[25] Austin S.A., Santhanam A.V., Katusic Z.S. (2010). Endothelial Nitric Oxide Modulates Expression and Processing of Amyloid Precursor ProteinNovelty and Significance. Circ. Res..

[26] Haul S., Gödecke A., Schrader J., Haas H.L., Luhmann H.J. (1999). Impairment of neocortical long-term potentiation in mice deficient of endothelial nitric oxide synthase. J. Neurophysiol..

[27] Austin S.A., D’Uscio L.V., Katusic Z.S. (2013). Supplementation of nitric oxide attenuates AβPP and BACE1 protein in cerebral microcirculation of eNOS-deficient mice. J. Alzheimers Dis..

[28] Howell S., Nalbantoglu J., Crine P. (1995). Neutral endopeptidase can hydrolyze β-amyloid(1–40) but shows no effect on β-amyloid precursor protein metabolism. Peptides.

[29] Takaki Y., Iwata N., Tsubuki S., Taniguchi S., Toyoshima S., Lu B., Gerard N.P., Gerard C., Lee H.J., Shirotani K. (2000). Biochemical identification of the neutral endopeptidase family member responsible for the catabolism of amyloid beta peptide in the brain. J. Biochem..

[30] Colciaghi F., Borroni B., Zimmermann M., Bellone C., Longhi A., Padovani A., Cattabeni F., Christen Y., Di L.M. (2004). Amyloid precursor protein metabolism is regulated toward alpha-secretase pathway by Ginkgo biloba extracts. Neurobiol. Dis..

[31] Baker L.D., Cross D.J., Minoshima S., Belongia D., Watson G.S., Craft S. (2011). Insulin resistance and Alzheimer-like reductions in regional cerebral glucose metabolism for cognitively normal adults with prediabetes or early type 2 diabetes. Arch. Neurol..

[32] Subhadra B., Schaller K., Seeds N.W. (2013). Neuroserpin up-regulation in the Alzheimer’s disease brain is associated with elevated thyroid hormone receptor-β1 and HuD expression. Neurochem. Int..

[33] Yan R., Vassar R. (2014). Targeting the β secretase BACE1 for Alzheimer’s disease therapy. Lancet Neurol..

[34] Vassar R., Kovacs D.M., Yan R., Wong P.C. (2009). The β-Secretase Enzyme BACE in Health and Alzheimer’s Disease: Regulation, Cell Biology, Function, and Therapeutic Potential. J. Neurosci..

[35] White H.L., Scates P.W., Cooper B.R. (1996). Extracts of Ginkgo biloba leaves inhibit monoamine oxidase. Life Sci..

[36] Pardon M.C., Joubert C., Perez-Diaz F., Christen Y., Launay J.M., Cohen-Salmon C. (2000). In vivo regulation of cerebral monoamine oxidase activity in senescent controls and chronically stressed mice by long-term treatment with Ginkgo biloba extract (EGb 761). Mech. Aging Dev..

[37] Chaurasiya N.D., Ganesan S., Nanayakkara N.P., Dias L.R., Walker L.A., Tekwani B.L. (2012). Inhibition of human monoamine oxidase A and B by 5-phenoxy 8-aminoquinoline analogs. Bioorg. Med. Chem. Lett..

[38] Aliev G., Seyidova D., Lamb B.T., Obrenovich M.E., Siedlak S.L., Vinters H.V., Friedland R.P., Lamanna J.C., Smith M.A., Perry G. (2003). Mitochondria and vascular lesions as a central target for the development of Alzheimer’s disease and Alzheimer disease-like pathology in transgenic mice. Neurol. Res..

[39] Biere A.L., Ostaszewski B., Stimson E.R., Hyman B.T., Maggio J.E., Selkoe D.J. (1996). Amyloid beta-peptide is transported on lipoproteins and albumin in human plasma. J. Biol. Chem..

[40] Li G.H., Arora P.D., Chen Y., Mcculloch C.A., Liu P. (2012). Multifunctional roles of gelsolin in health and diseases. Med. Res. Rev..

[41] Atwood C.S., Meethal S.V., Liu T., Wilson A.C., Gallego M., Smith M.A., Bowen R.L. (2005). Dysregulation of the hypothalamic-pituitary-gonadal axis with menopause and andropause promotes neurodegenerative senescence. J. Neuropathol. Exp. Neurol..

[42] Meethal S.V., Smith M.A., Bowen R.L., Atwood C.S. (2005). The gonadotropin connection in Alzheimer’s disease. Endocrine.

[43] Pedrós I., Petrov D., Artiach G., Abad S., Ramon-Duaso C., Sureda F., Pallàs M., Beas-Zarate C., Folch J., Camins A. (2015). Adipokine pathways are altered in hippocampus of an experimental mouse model of Alzheimer’s disease. J. Nutr. Health Aging.

[44] Dye R.V., Miller K.J., Singer E.J., Levine A.J. (2012). Hormone Replacement Therapy and Risk for Neurodegenerative Diseases. Int. J. Alzheimers Dis..

[45] Long J., He P., Shen Y., Li R. (2012). New evidence of mitochondria dysfunction in the female Alzheimer’s brain: Deficiency of estrogen receptor-β. J. Alzheimers Dis..

[46] Jackson H.M., Soto I., Graham L.C., Carter G.W., Howell G.R. (2013). Clustering of transcriptional profiles identifies changes to insulin signaling as an early event in a mouse model of Alzheimer’s disease. BMC Genom..

[47] Burbach J.P., Voorhuis T.A., van Tol H.H., Ivell R. (1987). In situ hybridization of oxytocin messenger RNA: Macroscopic distribution and quantitation in rat hypothalamic cell groups. Biochem. Biophys. Res. Commun..

[48] Hollowell J.G., Staehling N.W., Flanders W.D., Hannon W.H., Gunter E.W., Spencer C.A., Braverman L.E. (2002). Serum TSH, T(4), and thyroid antibodies in the United States population (1988 to 1994): National Health and Nutrition Examination Survey (NHANES III). J. Clin. Endocr. Metab..

[49] Riederer P., Bartl J., Laux G., Grünblatt E. (2011). Diabetes type II: A risk factor for depression-Parkinson-Alzheimer?. Neurotox. Res..

[50] Huang S.M., Mouri A., Kokubo H., Nakajima R., Suemoto T., Higuchi M., Staufenbiel M., Noda Y., Yamaguchi H., Nabeshima T. (2006). Neprilysin-sensitive synapse-associated amyloid-beta peptide oligomers impair neuronal plasticity and cognitive function. J. Biol. Chem..

[51] Zhao L., Yao J., Mao Z., Chen S., Wang Y., Brinton R.D. (2011). 17β-Estradiol regulates insulin-degrading enzyme expression via an ERβ/PI3-K pathway in hippocampus: Relevance to Alzheimer’s prevention. Neurobiol. Aging.

[52] Wang L., Andersson S., Warner M., Gustafsson J.A. (2001). Morphological abnormalities in the brains of estrogen receptor beta knockout mice. Proc. Natl. Acad. Sci. USA.

[53] Waters E.M., Yildirim M., Janssen W.G.M., Lou W.Y.W., Mcewen B.S., Morrison J.H., Milner T.A. (2011). Estrogen and aging affect the synaptic distribution of estrogen receptor α-immunoreactivity in the CA1 region of female rat hippocampus. Brain Res..

[54] Zhao L., Mao Z., Schneider L.S., Brinton R.D. (2011). Estrogen receptor β-selective phytoestrogenic formulation prevents physical and neurological changes in a preclinical model of human menopause. Menopause.

[55] Kalgutkar A.S., Dalvie D.K., Castagnoli N., Taylor T.J. (2001). Interactions of nitrogen-containing xenobiotics with monoamine oxidase (MAO) isozymes A and B: SAR studies on MAO substrates and inhibitors. Chem. Res. Toxicol..

[56] Arai T., Miklossy J., Klegeris A., Guo J.P., Mcgeer P.L. (2006). Thrombin and prothrombin are expressed by neurons and glial cells and accumulate in neurofibrillary tangles in Alzheimer disease brain. J. Neuropathol. Exp. Neurol..

[57] Era S., Kuwata K., Imai H., Nakamura K., Hayashi T., Sogami M. (1995). Age-related change in redox state of human serum albumin. Biochim. Biophys. Acta.

[58] Guerin-Dubourg A., Catan A., Bourdon E., Rondeau P. (2012). Structural modifications of human albumin in diabetes. Diabetes Metab..

[59] Johansson P., Almqvist E.G., Johansson J.O., Mattsson N., Hansson O., Wallin A., Blennow K., Zetterberg H., Svensson J. (2013). Reduced cerebrospinal fluid level of thyroxine in patients with Alzheimer’s disease. Psychoneuroendocrinology.

[60] Ray I., Chauhan A., Wegiel J., Chauhan V. (2000). Gelsolin inhibits the fibrillization of amyloid beta-protein, and also defibrillizes its preformed fibrils. Brain Res..

[61] Hirko A.C., Meyer E.M., King M.A., Hughes J.A. (2007). Peripheral Transgene Expression of Plasma Gelsolin Reduces Amyloid in Transgenic Mouse Models of Alzheimer’s Disease. Mol. Ther. J. Am. Soc. Gene Ther..

